# Supplementary figures and images for: Biochemical and molecular responses of maize to low and high temperatures in symbiosis with mixed arbuscular mycorrhizal fungi cultures
Source: PeerJ. 2025 Nov 28;13:e20419. doi: 10.7717/peerj.20419 (PMC12667686; doi:10.7717/peerj.20419)

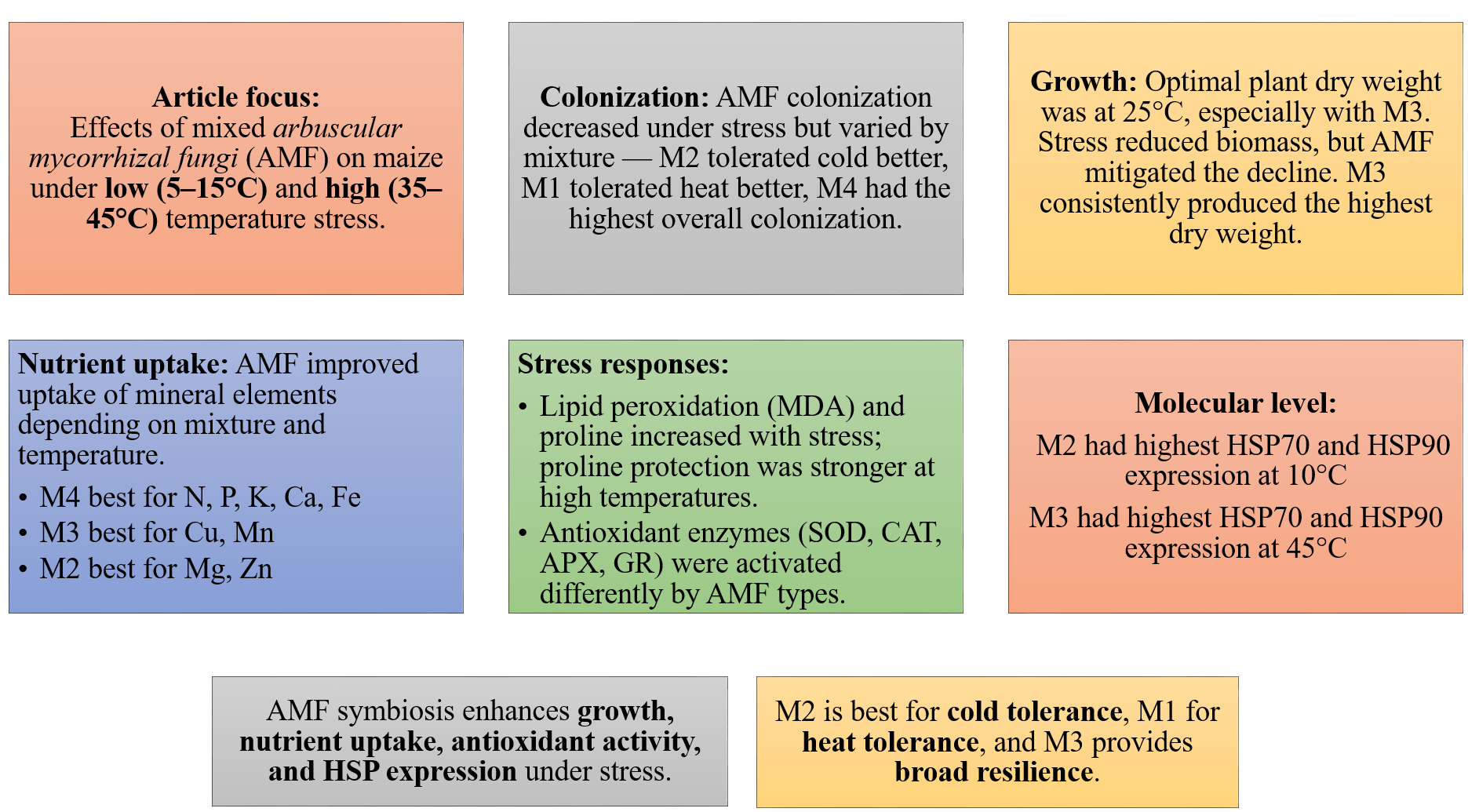

Supplement: Supplemental Information 2 [file peerj-13-20419-s002.png]
